# Differential neuroprotective potential of CRMP2 peptide aptamers conjugated to cationic, hydrophobic, and amphipathic cell penetrating peptides

**DOI:** 10.3389/fncel.2014.00471

**Published:** 2015-01-26

**Authors:** Aubin Moutal, Liberty François-Moutal, Joel M. Brittain, May Khanna, Rajesh Khanna

**Affiliations:** ^1^Department of Pharmacology, College of Medicine, University of ArizonaTucson, AZ, USA; ^2^Paul and Carole Stark Neurosciences Research Institute, Indiana University School of MedicineIndianapolis, IN, USA; ^3^Neuroscience Graduate Interdisciplinary Program, College of Medicine, University of ArizonaTucson, AZ, USA

**Keywords:** NMDAR, CRMP2, neuroprotection, cell-penetrating peptide, delayed calcium dysregulation, excitotoxicity

## Abstract

The microtubule-associated axonal specification collapsin response mediator protein 2 (CRMP2) is a novel target for neuroprotection. A CRMP2 peptide (TAT-CBD3) conjugated to the HIV transactivator of transcription (TAT) protein’s cationic cell penetrating peptide (CPP) motif protected neurons in the face of toxic levels of Ca^2+^ influx leaked in via *N*-methyl-D-aspartate receptor (NMDAR) hyperactivation. Here we tested whether replacing the hydrophilic TAT motif with alternative cationic (nona-arginine (R9)), hydrophobic (membrane transport sequence (MTS) of k-fibroblast growth factor) or amphipathic (model amphipathic peptide (MAP)) CPPs could be superior to the neuroprotection bestowed by TAT-CBD3. In giant plasma membrane vesicles (GPMVs) derived from cortical neurons, the peptides translocated across plasma membranes with similar efficiencies. Cortical neurons, acutely treated with peptides prior to a toxic glutamate challenge, demonstrated enhanced efflux of R9-CBD3 compared to others. R9-CBD3 inhibited *N*-methyl-D-aspartate (NMDA)-evoked Ca^2+^ influx to a similar extent as TAT-CBD3 while MTS-CBD3 was ineffective which correlated with the ability of R9- and TAT-CBD3, but not MTS-CBD3, to block NMDAR interaction with CRMP2. Unrestricted Ca^2+^ influx through NMDARs leading to delayed calcium dysregulation and neuronal cell death was blocked by all peptides but MAP-CBD3. When applied acutely for 10 min, R9-CBD3 was more effective than TAT-CBD3 at neuroprotection while MTS- and MAP-CBD3 were ineffective. In contrast, long-term (>24 h) treatment with MTS-CBD3 conferred neuroprotection where TAT-CBD3 failed. Neither peptide altered surface trafficking of NMDARs. Neuroprotection conferred by MTS-CBD3 peptide is likely due to its increased uptake coupled with decreased efflux when compared to TAT-CBD3. Overall, our results demonstrate that altering CPPs can bestow differential neuroprotective potential onto the CBD3 cargo.

## Introduction

Excitotoxicity is characterized as a pathological process by which a disproportionate exposure to the neurotransmitter glutamate leads to an overstimulation of its cognate membrane receptors. This results in a disruption in cell membrane permeability, downstream activation of signaling cascades involved in loss of nerve cell function, culminating in cell death (Gillessen et al., 2002; Lau and Tymianski, 2010). An early event in the sequelae leading to excitotoxicity is the excessive activation of *N*-methyl-D-aspartate (NMDA)-type glutamate receptors resulting in a massive influx of Ca^2+^, making NMDAR a much sought after target for prevention of excitotoxicity (Faden et al., 1989; Grotta et al., 1990; Steinberg et al., 1995). Moreover, NMDAR has a number of sites that have been exploited pharmacologically, including the ion channel pore, the glutamate-binding site, the glycine-binding site and the polyamine interaction site, but since NMDAR activity is crucial for normal neuronal function, the efforts to develop NMDAR antagonists have unequivocally failed in clinical trials due to their toxicity (Ikonomidou et al., 2000; Ikonomidou and Turski, 2002; Muir, 2006). In this context, targeting the proteins regulating the NMDAR may offer an advantage in preventing excitotoxicity with the possibility of minimal side effects.

Among the multitude of NMDAR associated proteins (Al-Hallaq et al., 2007), the microtubule-associated collapsin response mediator protein 2 (CRMP2), generally known for its role in growth cone collapse in sensory neurons (Goshima et al., 1995), has been reported as a possible regulator of NMDAR activity and localization (Bretin et al., 2006; Al-Hallaq et al., 2007). Following glutamate excitotoxicity, CRMP2 expression is decreased and this down-regulation is associated with increased axonal injury and loss, implicating CRMP2 in neuronal death mechanisms (Xiong et al., 2012). Further evidence of a putative link between CRMP2 and NMDAR can be surmised from studies on CRMP2 and neurotoxicity that reported calcium-activated protease calpain-mediated cleavage of CRMP2 occurring in response to sundry neurotoxic insults including injury, ischemia, and excitotoxic exposure to glutamate (Chung et al., 2005; Bretin et al., 2006; Jiang et al., 2007; Touma et al., 2007; Hou et al., 2009; Zhang et al., 2009). CRMP2 is cleaved following glutamate exposure; neurons expressing the calpain-cleaved form of CRMP2 had reduced NMDAR responses and decreased neurotoxicity. Conversely, overexpression of CRMP2 was neurotoxic. This suggested that cleaved CRMP2 has a dominant negative effect leading to enhanced neuronal survival (Bretin et al., 2006). Furthermore, NMDAR surface expression was reportedly decreased when calpain-cleaved CRMP2 was overexpressed. These findings supported the notion that calpain-cleaved CRMP2 may be neuroprotective by reducing NMDAR surface expression and, by inference, that overexpression of CRMP2 is perhaps neurotoxic by upregulating NMDAR surface expression. Another study linking CRMP2 to NMDARs reported a biochemical complex between CRMP2 and NR2A and NR2B subunits of NMDARs (Al-Hallaq et al., 2007).

Recent studies have highlighted the use of CRMP2 peptide aptamers for targeting of both ligand- and voltage-gated Ca^2+^ channels (Brittain et al., 2011a,b, 2012). Importantly, the Ca^2+^-channel binding domain 3 (CBD3) peptide of CRMP2 has also been shown to reduce various pain states (Brittain et al., 2011b; Wilson et al., 2011, 2012; Piekarz et al., 2012; Ripsch et al., 2012) as well as enhance neuronal survival following cerebral ischemia and traumatic brain injury. Mechanistically, it appears that CBD3 is able to enhance neuronal survival through direct inhibition of NMDARs (Brittain et al., 2011a, 2012). In addition we observed that application of transactivator of transcription domain (TAT)-CBD3 led to a dendritic specific reduction of NR2B NMDAR subunit surface expression. These findings suggest that TAT-CBD3 may have a dual mode of action on NMDARs. It is unknown if these two modes of action converge mechanistically and if a CBD3 peptide could be developed which inhibits NMDAR activity without changing surface expression.

In the current study we sought to develop new CBD3 peptides through coupling CBD3 to cell penetrating peptides (CPPs) with different properties. The rationale of this facile approach was that attaching a different CPP might generate a CBD3 that has properties distinct from the previously characterized TAT-CBD3. It has been generally assumed that the CPP is itself inert, although evidence to the contrary is beginning to emerge (Brugnano et al., 2010). For example, it has been reported that the CPPs themselves can inhibit proteolytic activity (Cameron et al., 2000; Horn et al., 2000; Fugere et al., 2007; Kloss et al., 2009), modulate the metabolic profile of cells (Kilk et al., 2009), alter gene expression (Kuo et al., 2009), and inhibit kinase activity (Ward et al., 2009). What can be surmised from these findings is that the biological activity is related to the CPP sequence and it is imperative to examine a range of CPPs with any cargo to rule out unintended biological activities. Here, we chose the CPPs nona-arginine (R9), the model amphipathic peptide (MAP), and the membrane translocating sequence (MTS) of Kaposi fibroblast growth factor (k-FGF) receptor as the representatives of different subclasses of CPPs—cationic (R9), primary amphipathic CPPs (MTS), and secondary amphipathic α-helical CPPs (MAP) and compared them to TAT, a primary hydrophilic charged CPP.

We find that CBD3-mediated inhibition of NMDARs is dependent upon the CPP that it is attached to. Furthermore, we observed that attaching the MTS CPP to CBD3 generates a peptide that is neuroprotective following prolonged, but not acute, application.

## Materials and methods

### Animals

Pathogen-free, pregnant Sprague-Dawley rats (150–200 g) were purchased from Harlan Laboratories (Madison, WI, USA). The Institutional Animal Care and Use of the Indiana University School of medicine and the College of Medicine at the University of Arizona Committees approved these experiments. All procedures were conducted in accordance with the Guide for Care and Use of Laboratory Animals published by the National Institutes of Health and the ethical guidelines of the International Association for the Study of Pain.

### Peptides

All peptides were synthesized by Genscript Inc. (Piscataway, NJ, USA) or Covalab (Villeurbanne, France) and verified by mass spectrometry prior to use. The peptide sequences are as follows: CBD3: ARSRLAELRGVPRGL; MAP: KLALKLALKALKAALKLA; MTS: AAVALLPAVLLALLAP; TAT: YGRKKRRQRRR and R9: RRRRRRRRR. Peptide stock concentrations of 20 mM were made in water and stored at −80°C in single-use aliquots. CBD3 was conjugated C-terminal to the CPPs. Fluorescein isothiocyanate (FITC)-labeled CBD3 peptides with the fluorescent label at the N-terminus were also purchased.

### Embryonic cortical neuron cultures

Cortical neuron cultures were prepared from embryonic day 19 (E19) Sprague-Dawley rat pups as previously described for hippocampal neurons (Brittain et al., 2009). Neurons were grown for 7–8 days *in vitro* (DIV) prior to experiments.

### Formation of giant plasma membrane vesicles (GPMVs)

Giant plasma membrane vesicles (GPMVs) were obtained as previously described in Sezgin et al. (2012). Briefly, GPMVs were generated from cortical neurons incubated at 37°C for 90 min in a vesiculation buffer containing 10 mM HEPES, 150 mM NaCl, 2 mM CaCl_2_, pH 7.4 containing 2 mM *N*-ethyl maleimide (NEM). After GPMV formation, the buffer containing vesicles was recovered and conserved at 4°C for at least 2 h to allow the vesicles to concentrate at the bottom of the tube. As the GPMVs were stable for ~48 h (data not shown), they were used the day after formation at the latest.

### Peptide–GPMV incubation and labeling

All experiments for microscopic analyses of GPMVs were performed on 35 mm glass bottom dishes pre-coated with BSA (1 mg/mL for 1 h at room temperature). Approximately 100 µL of GMPVs labeled with 5 µM di-4-ANEPPDHQ were incubated for 3 h at room temperature with 10 µM of CBD3 peptide aptamers. di-4-ANEPPDHQ, a styryl dye that was originally developed to detect transmembrane potential changes, is considered to be a good probe for rafts (Jin et al., 2005) and was used to label GPMVs. di-4-ANEPPDHQ is able to partition into both liquid-ordered and liquid-disordered phase domains in model membranes, reacts to the environmental difference between the two phases via conformational changes, thus resulting in different fluorescence properties (Jin et al., 2005). Here, we studied the 500–550 nm emission of the probe, which accounts largely for the liquid ordered phase.

### Fluorescence microscopy and image analysis

Fluorescence imaging was performed with an inverted microscope, Nikon Eclipse TE2000-U, using objective Nikon Super Fluor 20× 0.75 NA and a Photometrics cooled CCD camera CoolSNAPHQ-ES2 (Roper Scientific, Tucson, AZ, USA) controlled by MetaFluor 6.3 software (Molecular Devices, Downingtown, PA, USA). The excitation light was delivered by a Lambda-LS system (Sutter Instruments, Novato, CA, USA). The excitation filters were controlled by a Lambda 10-2 optical filter change (Sutter Instruments). Twenty images of each condition were systematically recorded randomly, using a FITC filter (excitation and emission wavelength 488 nm and 500–550 nm, respectively), which accounted for the liquid ordered contribution of di-4-ANEPPDHQ or the localization of FITC-peptides.

### Uptake and efflux of CBD3 peptides from cortical neurons

Cortical neurons were seeded into 96-well plates at 3.5 × 10^4^ cells per well and cultured until 7 DIV at 37°C and 5% CO_2_. 20 µM of FITC conjugated CBD3 peptide aptamers were added on neurons and allowed to penetrate the cells for 10 min. After four washes with phenol red free Leibovitz medium, the FITC fluorescence in the cells was measured using a Synergy 2 fluorescent plate reader (Biotek, Winooski, VT, USA) at an excitation wavelength of 485 nm and emission wavelength of 530 nm. Efflux of the peptides from the cells was assessed by measuring FITC fluorescence in the media after 0, 10, 30 and 60 min. To correct for any differences in cell plating, at the end of the experiment cortical neurons were lysed with 20 mM Tris, pH 7.4, 50 mM NaCl, 1% NP40, 0.5% sodium deoxycholate, 0.1% SDS Protease inhibitor cocktail set III (Calbiochem), phosphatase inhibitor cocktail set I (Calbiochem), 50 U/ml benzonase (Merck) and the protein quantity determined with a Pierce assay.

### Co-immunoprecipitation

Rat brains were lysed into the immunoprecipitation buffer containing 20 mM Tris-HCl pH 7.4, 50 mM NaCl, 2 mM MgCl_2_, 1% (vol/vol) NP40, 0.5% (mass/vol) sodium deoxycholate with Protease/phosphatase inhibitors cocktails (Calbiochem) and Benzonase (50 U/mL^−1^), using a dounce homogenizer. The lysates were clarified by centrifugation at 10000 g, 10 min, 4°C then total protein concentration was determined by BCA protein assay (Cat# PI23225, Thermo scientific). For the co-immunoprecipitation, 300 µg of total protein was incubated with 1 µg of CRMP2 antibody (Cat# C2993, Sigma, St. Louis, MO, USA) of non-specific rabbit IgG, in the presence of 10 µM of the indicated peptides and incubated overnight at 4°C under gentle agitation. Protein G magnetic beads (Cat# 10004D, Life Technologies) pre-equilibrated with the immunoprecipitation buffer, were added to the mixture and allowed to incubate for 1 h at 4°C to capture the immuno-complexes. The beads were washed four times with the immunoprecipitation buffer before re-suspension in Laemmli buffer and boiling at 95°C for 5 min prior to immunoblotting.

### Immunoblot analysis

Indicated samples were loaded on 4–12% NuPAGE® gels (Life Technologies). Proteins were transferred to polyvinylidene difluoride membranes preactivated in methanol and blocked at room temperature for 1 h with TBST containing 5% non-fat dry milk. The saturated membranes were incubated separately in TBST containing 5% BSA with the primary antibodies CRMP2 (Cat# C2993, Sigma, St. Louis, MO, USA), NR2B (Cat# 610416, BD biosciences, San Jose, CA, USA) or Kv2.1 (Cat# K89/34, NeuroMab, Davis, CA, USA) overnight at 4°C. Following incubation in horseradish peroxidase conjugated secondary antibodies (Jackson immunoresearch), blots were revealed by enhanced luminescence (WBKLS0500, Millipore) before exposure to photographic film. Films were scanned, and quantified using Un-Scan-It gel version 6.1 scanning software (Silk Scientific Inc, Orem, UT, USA).

### Calcium imaging

NMDAR-mediated Ca^2+^ influx was monitored as the ratio of F340/F380 using the ratiometric Ca^2+^ dye Fura-2 as previously described with minor modifications (Brittain et al., 2011a). Neurons were loaded with 3 µM Fura-2 AM in extracellular bath solution (139 mM NaCl, 3 mM KCl, 0.8 mM MgCl_2_, 1.8 mM CaCl_2_, 10 mM NaHEPES, pH 7.4, 5 mM glucose) for 25 min at 37°C, 200 nM tetrodotoxin was also included to prevent action potentials. A baseline of at least six images (at 0.2 Hz) was collected prior to stimulation of neurons with 50 µM NMDA/ 20 µM glycine in MgCl_2_-free buffer (to prevent Mg^2+^ block of NMDARs). After 10 s NMDA/glycine was removed and neurons were bathed in MgCl_2_-containing buffer. Only cells that displayed a greater than 50% increase compared to baseline were used for subsequent analyses.

For delayed calcium deregulation experiments, neurons pre-treated for 10 min with 10 µM of the indicated peptides were loaded with 2.6 µM Fura-2FF-AM for 30 min at 37°C. A baseline was acquired before stimulation with 25 µM glutamate plus 10 µM glycine. The peptides remained in the bath solution throughout the experiment. To minimize photobleaching and phototoxicity, the images were taken every ~10 s during the time-course of the experiment using the minimal exposure time that provided acceptable image quality.

### Glutamate-induced toxicity

E19 neurons (grown in 96 well plates) were stimulated with 200 µM glutamate/20 µM glycine to induce neuronal death as previously described with modifications (Brittain et al., 2011a). Neurons were pre-treated with peptides by removing half of the culture medium and replacing with fresh medium containing twice the final concentration of peptide. Neurons were then stimulated by removing half the medium and replacing with fresh medium containing 400 µM glutamate/40 µM glycine and 1X peptide concentration. Following a 30 min stimulation the medium was completely removed and replaced with half fresh/half conditioned medium. Cell viability was quantified 24 h later using the 3-(4,5-dimethylthiazol-2-yl)-5-(3-carboxymethoxyphenyl)-2-(4-sulfophenyl)-2H-tetrazolium cell viability assay (Promega, Madison, WI, USA) as previously described (Brittain et al., 2011a). Subunit-specific NMDAR antagonists Peaqx (NR2A) and Ifenprodil (NR2B) were purchased from Sigma.

### Biotinylation of cortical neurons

E19 neurons were biotinylated and neuronal lysates were generated and probed by Western blotting as previously described (Brittain et al., 2011a).

### Statistics

Average values with standard errors of the mean are presented. Samples were considered to be statistically significant if *p* < 0.05 using a one-way ANOVA with Dunnett’s *post hoc* for comparisons with more than three conditions or using Student’s *t*-test for comparison of three conditions or less.

## Results

### Rationale for selection of cationic and amphipathic cell penetrating peptides (CPPs)

We recently reported that CRMP2 knockdown is neuroprotective (Brittain et al., 2011a) and that the CRMP2 peptide, TAT-CBD3, is able to prevent neuronal cell death from toxic glutamate exposure via inhibition of NMDAR-mediated Ca^2+^-influx (Brittain et al., 2011a, 2012). Based on these studies, here we sought to test if other natural and synthetic CPP motifs fused to CBD3 could be more effective than TAT-CBD3 in reducing glutamate-mediated toxicity. Three additional CPPs were selected: (i) the nona-arginine (R9) which has a half-life (*t*_1/2_) of ~2 h (Sarko et al., 2010) and a greater than 20-fold penetration compared to TAT (Wender et al., 2000) using macropinocytosis; (ii) the α-helical MAP with a *t*_1/2_ of >72 h (Sarko et al., 2010) which translocates cargo into cells in a non-endocytic fashion using multiple non-specific, energy-dependent and -independent processes (Oehlke et al., 1998); and (iii) the MTS of k-FGF receptor with a *t*_1/2_ of ~48 h (Sarko et al., 2010) which has a hydrophobic stretch of residues necessary for import into cells that likely occurs via a non-endocytotic route using an energy- and temperature-independent translocation process reliant on its interactions with the membrane (Lin et al., 1995). These were compared to TAT, which has a *t*_1/2_ of ~9 h (Sarko et al., 2010) and is posited to enter into cells via macropinocytosis, energy- and temperature-independent pathways, as well as endocytic uptake mechanisms (see review by Torchilin and colleagues (Sawant et al., 2013).

### Interrogating membrane penetration of peptides using giant plasma membrane vesicles (GPMVs) of cortical neurons

**Figure 1:**
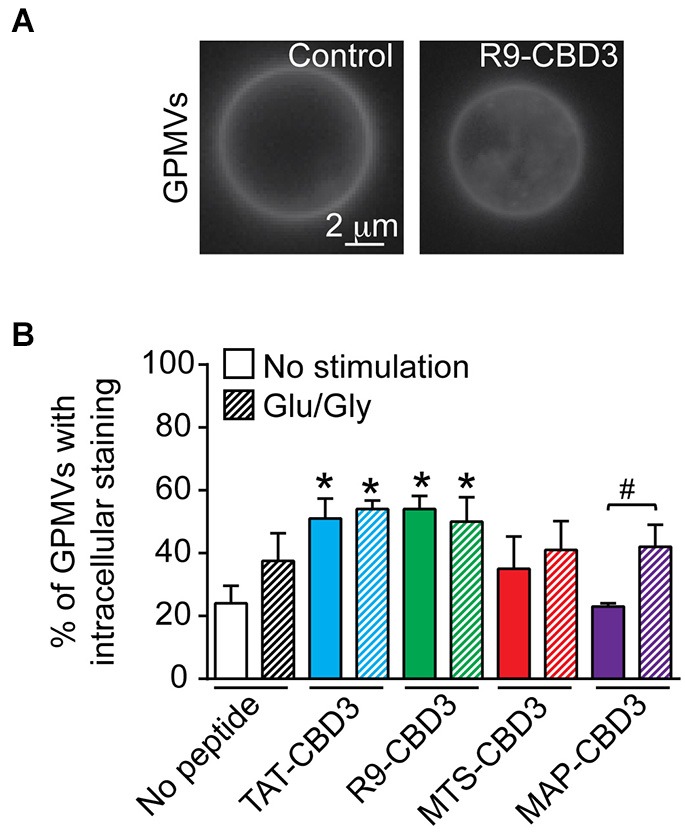
**Differential intracellular accumulation of CBD3 peptides conjugated to cationic and amphipathic CPPs in model giant plasma membrane vesicles (GPMVs) from cortical neurons**. GPMVs derived from cortical neurons were incubated with the fluorescent membrane-staining dye, di-4-ANEPPDHQ (5 µM), in the absence (control) or presence of the indicated peptides (10 µM) for 60 min at room temperature. **(A)** Representative fluorescence (pseudocolor black and white) images of di-4-ANEPPDHQ-stained control- or R9-CBD3-treated GPMVs. Scale bar: 2 µm. **(B)** Percent of di-4-ANEPPDHQ-stained control- or peptide-treated GPMVs exhibiting lumenal accumulation. Shown are vesicles prepared from untreated cortical neurons as well as neurons challenged for 1 h with 200 µM glutamate + 20 µM glycine (Glu/Gly) prior to the vesiculation. *p < 0.01 comparing GPMVs from control, unstimulated cells vs. TAT- or R9-CBD3-treated cortical neurons irrespective of Glu/Gly challenge. *#p* < 0.01 comparing GPMVs from control vs. Glu/Gly-challenged cells treated with MAP-CBD3. *n* = 2 separate, individual experiments from separate cortical culture preparations; the total number of GPMVs analyzed is 568.

We began by testing if the natural and synthetic CPPs bestowed differential penetration to CBD3. To avoid any issues possibly arising from fluorescent labeling of peptides, we resorted to the use of GPMVs, which are a useful model to study peptide-lipid dynamics as well as the translocation of PTDs across the plasma membrane (Saalik et al., 2011; Maniti et al., 2014). GPMVs represent a natural membrane model system with a cytoplasmic lumen devoid of cellular organelles and the actin cytoskeleton, low intracellular membrane content, and mimic the protein and lipid composition of the plasma membrane, having a phospholipid/cholesterol ratio of ~2:1 (Fridriksson et al., 1999; Charras et al., 2005; Bauer et al., 2009). Following chemically-induced vesiculation with NEM (Sezgin et al., 2012), cortical neurons released GPMVs. Isolated GPMVs typically segregate their lipids into a liquid-ordered phase composed of tightly packed, cholesterol and sphingolipids, reminiscent of raft-like domains (Fridriksson et al., 1999); di-4-ANEPPDHQ, a styryl dye that was originally developed to detect transmembrane potential changes, is considered to be a good probe for rafts (Jin et al., 2005). di-4-ANEPPDHQ-labeled GPMVs had a spherical shape and varied in size from ~3 to 13 µm in diameter. Following incubation with 5 µM di-4-ANEPPDHQ, the membranes of the majority of the GPMVs revealed an optically homogeneous lipid phase in untreated (control) and peptide-treated (Figure 1A) conditions. Incubation with CPP-conjugated CBD3 peptides resulted in a presumptive loss in the integrity of the membrane resulting in the fragmentation of some GPMVs into smaller vesicles that accumulated within the larger GPMVs; between ~34 (for MAP-CBD3) to ~54% (for TAT- or R9-CBD3) of GPMVs exhibited this phenotype, far greater than untreated- or MAP-CBD3-treated GPMVs vesiculated from control cortical cells (Figure 1B). Next, we tested if GPMVs made from cortical neurons following glutamate-induced toxicity had altered membrane penetration/distribution. GPMVs from Glu/Gly-treated neurons did not differ in their shape or size from control neurons. The percent of GPMVs with intracellular accumulation of the di-4-ANEPPDHQ dye were 2.1- to 2.6-fold greater than GMPVS from control untreated cells (Figure 1B). While the excitotoxic challenge did not alter the extent of penetration of TAT-, R9-, and MTS-conjugated CBD3 peptides, the percent of GPMVs with intracellular staining following MAP-CBD3 incubation was ~45% greater in vesicles from cells with excitotoxic challenge than without (Figure 1B). Collectively, these results demonstrate that all CPPs conjugated to CBD3 have similar penetration potential into model plasma membranes.

### Influx and efflux of CBD3 peptides conjugated to cationic and amphipathic CPPs

**Figure 2:**
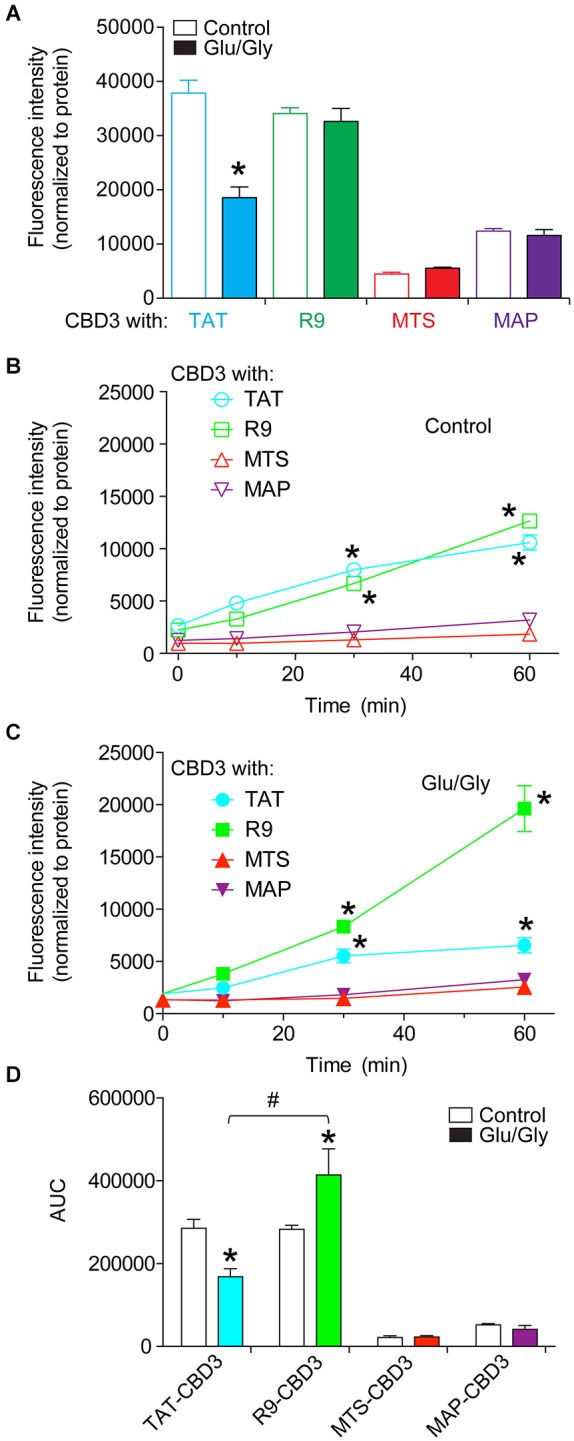
**Differential uptake and efflux of CBD3 peptides conjugated to cationic and amphipathic CPPs in cortical neurons**. Cortical neurons plated onto poly-D-lysine-coated 96-well plates were incubated with FITC-labeled CBD3 peptides (10 µM) for 10 min at 37°C, washed extensively with minimal essential media without phenol red, and fluorescence was measured using a fluorescent plate reader at an excitation wavelength of 485 nm and emission wavelength of 520 nm. **(A)** Mean fluorescence uptake of peptides into cortical neurons, normalized to the amount of protein per well, was similar between untreated and Glu/Gly challenged neurons for all peptides except TAT-CBD3, which was decreased in Glu/Gly challenged neurons compared to untreated neurons (*p < 0.01). Mean fluorescence efflux of peptides from untreated **(B)** or Glu/Gly-challenged **(C)** cortical neurons, normalized to the amount of protein per well, was significantly lower for MTS- and MAP-CBD3 at 10, 30 and 60 min compared to either TAT- or R9-CBD3 (*p < 0.01). Some error bars are smaller than the symbols. **(D)** Area under the curve (AUC) analyses reflecting cumulative efflux of the peptides in the indicated conditions. *p < 0.01 comparing AUC for TAT- or R9-CBD-treated control neurons vs. their respective Glu/Gly-challenged conditions. *#p* < 0.01 comparing AUC from stimulated cells with TAT-CBD3 vs. R9-CBD3-treated. *n* = 2 separate, individual experiments; the total number of wells analyzed is 8–13 per condition.

The varying propensities of the CBD3-conjugated peptides to segregate into lipid domains may contribute to their accumulation into and efflux from cortical neuron membranes. Therefore, we next quantitatively tested influx and rates of efflux of the peptides in cortical neurons without or following an excitotoxic challenge. FITC fluorescence was measured in cortical neurons following incubation with 20 µM fluorescently labeled CBD3 peptides. To minimize any possible variability in uptake due to differences in cell plating, we normalized the fluorescence per well to the amount of protein. The fluorescence intensities were not different between control- and Glu/Gly-treated neurons for all peptides except TAT-CBD3, which exhibited a significantly lower influx in cells challenged with glutamate toxicity (Figure 2A). Influx of MTS- and MAP-CBD3 peptides was less than that of TAT- and R9-CBD3-treated neurons irrespective of the excitotoxic challenge to the neurons (Figure 2A).

To address potential leakage of peptides from cortical neurons, the media of the neurons incubated with fluorescently labeled peptides was sampled immediately and 10, 30, and 60 min after peptide application. The fluorescence intensities were normalized to the amount of protein per well determined at the end of the experiment. At 30 and 60 min post peptide application, the fluorescence intensities recorded for TAT- and R9-CBD3-treated cells were greater than those for MTS- and MAP-CBD3-treated cells irrespective of the excitotoxic challenge (Figures 2B,C). The cumulative efflux, calculated from the area under the curve (AUC), was ~2.5-fold higher for R9-CBD3- vs. TAT-CBD3-treated cortical cells exposed to a glutamate challenge (Figure 2D).

### CRMP2-NR2B interaction can be differentially blocked by CBD3 peptides conjugated to cationic and amphipathic CPPs

**Figure 3:**
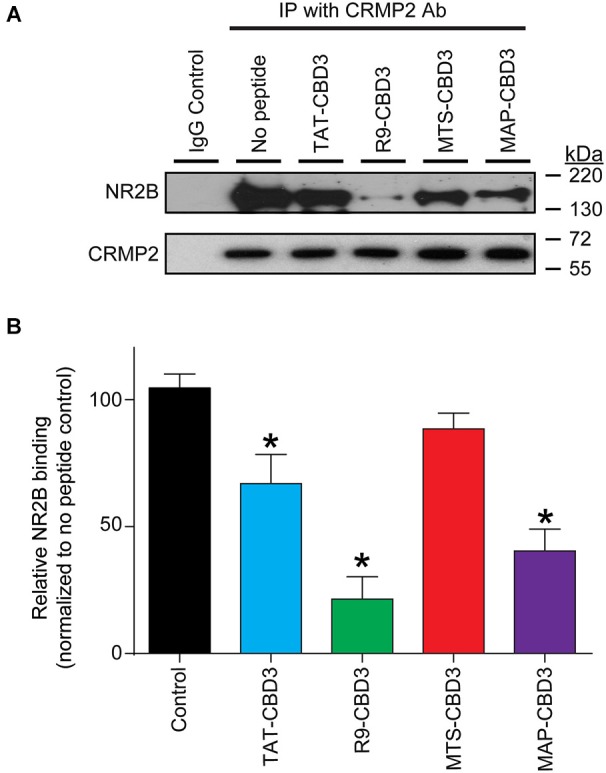
**Differential inhibition of the CRMP2 interaction with NR2B-NMDAR by CBD3 peptides conjugated to cationic and amphipathic CPPs**. **(A)** Lysates from rat brains were immunoprecipitated (*IP*) with antibodies against CRMP2 in the presence of 10 µM of the indicated peptides. The basal level of CRMP2-NR2B interaction was determined by immunoprecipitating the lysate without peptide and a non-specific IgG was used as a negative control. The immunoprecipitated complexes were immunoblotted with antibodies against NR2B (*top blot*) and CRMP2 (*bottom blot*). Representative blots from four separate experiments are shown. **(B)** Summary of the relative amount of NR2B bound to CRMP2 in the presence of the indicated peptides, normalized to the amount of immunoprecipitated CRMP2 and using the no peptide condition as a basal reference. *Asterisks* indicate statistical significance compared with untreated cells (*p* < 0.05, Kruskal-Wallis non-parametric test with a Dunnett’s *post hoc* analysis, *n* = 4).

Having established that the CPP-conjugated CBD3 peptides can enter cells, we next investigated if these peptides could recapitulate the previously reported uncoupling of the interaction between NR2B-NMDAR and CRMP2 (Brittain et al., 2012; Brustovetsky et al., 2014). Consistent with our previous findings (Brittain et al., 2012; Brustovetsky et al., 2014), co-immunoprecipitation experiments revealed an interaction between NR2B and CRMP2 in rat brain lysates (Figure 3A). TAT-CBD3 inhibited the NR2B-CRMP2 interaction by ~40% (Figures 3A,B; Xiong et al., 2012). R9- and MAP-CBD3 increased the extent of inhibition of the interaction with decreases of ~80% and ~60%, respectively, relative to no peptide control (Figures 3A,B). In contrast, MTS-CBD3 was ineffective in blocking the NR2B-CRMP2 interaction (Figures 3A,B). Although the amount of immunoprecipitated CRMP2 was slightly increased by both MTS- and MAP-CBD3 peptide treatments, the increases were not significant. These results demonstrate that, biochemically, use of different CPPs bestows a varying degree of inhibitory potential to the CBD3 cargo peptide.

### R9-CBD3 and MTS-CBD3 inhibit Ca^2+^-influx via NMDARs

**Figure 4:**
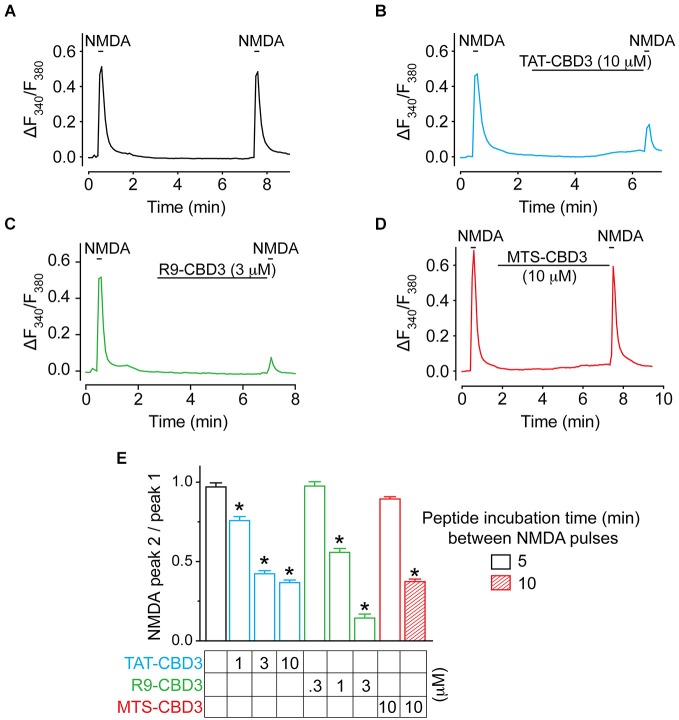
**TAT- and R9-CBD3, but not MTS-CBD3, inhibit NMDA-mediated Ca^2+^-influx. (A)** (Ca^2+^)_c_ was monitored in E18-19 DIV 7 cortical neurons using the Ca^2+^-sensitive dye Fura-2 following application of 50 µM NMDA + 100 µM glycine. Following application of NMDA, neurons were treated with vehicle (0.05% DMSO) for 10 min and then re-challenged with NMDA, before a final application ~5 min following the first one. **(B–D)** In addition to vehicle, neurons were also treated with either 10 µM TAT-CBD3, 10 µM R9-CBD3, or 10 µM MTS-CBD3 during the 5 min in the interim between the 1st and 2nd NMDA applications. A 10 min incubation between the two NMDA pulses was also tested for MTS-CBD3. **(E)** Bar graph summarizing the ratios of the 2nd NMDA application to the 1st for the various treatment conditions. *p < 0.05 compared to vehicle treated neurons (Kruskal-Wallis non-parametric test with a Dunnett’s *post hoc* analysis). Each value is from 2–4 experiments and at least 40 individual cells.

TAT-CBD3 reduces NMDAR-mediated Ca^2+^ influx (Brittain et al., 2011a), which likely explains the mechanism of its neuroprotection. Therefore, we first tested whether CBD3 fused to alternative CPPs could also inhibit NMDARs in a similar fashion. NMDAR-mediated Ca^2+^ influx was monitored using Fura-2 Ca^2+^ imaging in rat E19 cortical neurons cultured for DIV 7-8. Reproducible NMDAR-mediated peaks were observed following stimulation with 50 µM NMDA/20 µM glycine for 10 s (Figure 4A). Consistent with our previous findings (Brustovetsky et al., 2014), we observed robust inhibition of NMDAR in cells treated with 10 µM TAT-CBD3: peak 2 to peak 1 ratio (P2/P1) of 0.36 ± 0.02, indicating an ~64% inhibition of the peak NMDA response (Figures 4B,E). R9-CBD3 showed robust inhibition of NMDAR-mediated Ca^2+^ influx at 3 µM (Figures 4C,E). A 5 min incubation with 10 µM MTS-CBD3 displayed little inhibition of NMDARs as the P2/P1 ratio was not significantly different from control (Figures 4D,E). Slower internalization kinetics of the MTS CPP may possibly account for the lack of inhibition observed; therefore, a longer course of application of the peptide may be necessary. Indeed, incubating the peptide for 10 min after the first application of NMDA resulted in significant inhibition of NMDAR-mediated Ca^2+^ influx to almost the same extent as with a 5 min application of TAT-CBD3 peptide (Figure 4E). Application of 10 µM MAP-CBD3 led to a rise in Ca^2+^, which returned to background levels within 5 min (data not shown). This suggests MAP-CBD3 may be affecting membrane integrity through an unknown mechanism (but see Section Discussion) and may in fact be neurotoxic; for these reasons MAP-CBD3 was not tested further in these studies.

TAT- and R9-CBD3 were evaluated further using three concentrations of each (Figure 4E). At 3 µM, R9-CBD3 inhibited Ca^2+^ influx by ~85% inhibition compared to ~58% inhibition bestowed by TAT-CBD3 (Figure 4E). These results suggest that R9-CBD3 is a more potent inhibitor of NMDAR-mediated Ca^2+^ influx than TAT-CBD3 while MTS-CBD3 is also able to inhibit NMDARs but needs longer to do so.

### TAT-, R9-, and MTS-CBD3 peptides reduce glutamate-induced Ca^2+^ dysregulation

**Figure 5:**
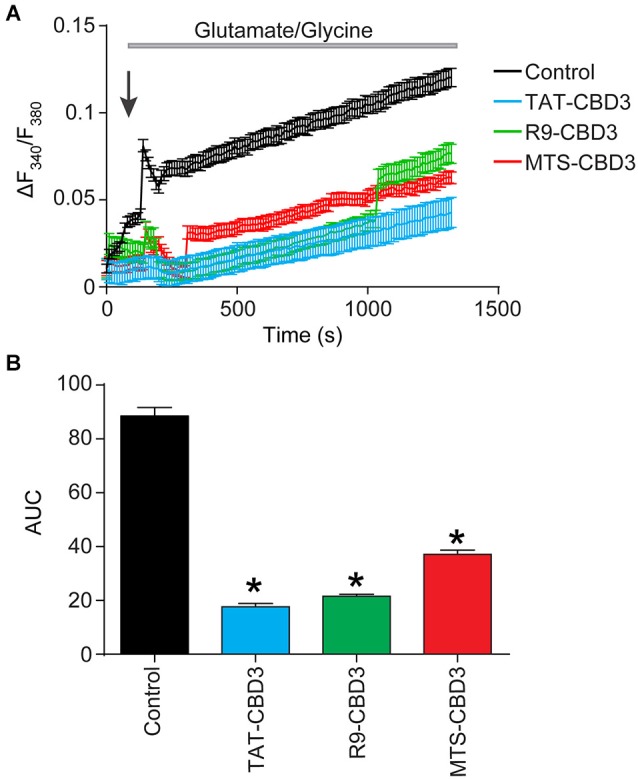
**TAT-, R9-, and MTS-CBD3 greatly reduce glutamate-induced DCD. (A)** [Ca^2+^]_c_ was monitored in E18–19 cortical neurons after prolonged exposure to 200 µM glutamate + 20 µM glycine using the Ca^2+^-sensitive Fura-2FF. Neurons were treated with vehicle (0.05% DMSO) or 10 µM of the peptides for 10 min prior to stimulation with Glu/Gly. *Arrow* indicated time of Glu/Gly application. **(B)** Bar graph displays the average AUC, in arbitrary units. Values represent *n* = 66–146 cells obtained from two separate experiments for each treatment. *Asterisks* indicate statistical significance compared with untreated cells (*p* < 0.05, Kruskal-Wallis non-parametric test with a Dunnett’s *post hoc* analysis).

We previously demonstrated that TAT-CBD3 significantly attenuated glutamate-induced delayed Ca^2+^ deregulation (DCD; Brittain et al., 2011a); DCD is a phenomenon whereby neurons accumulate toxic levels of intracellular Ca^2+^ due to the inability of the mitochondria to buffer the large Ca^2+^ overload following a prolonged exposure to glutamate (Nicholls, 2004). Neurons were incubated with vehicle (0.05% DMSO) or 10 µM CPP-CBD3 peptides for 10 min prior to a challenge with 200 µM glutamate + 20 µM glycine (Figure 5). This stimulation led to a sustained increase in (Ca^2+^)_c_ throughout the time course of the experiment. Compared to vehicle control, TAT-, R9-, and MTS-CBD3 peptides significantly reduced glutamate-induced DCD by ~80%, ~76%, and ~58%, respectively (Figures 5A,B).

### R9-CBD3 is more efficacious than TAT-CBD3 in preventing glutamate-induced neuronal death

**Figure 6:**
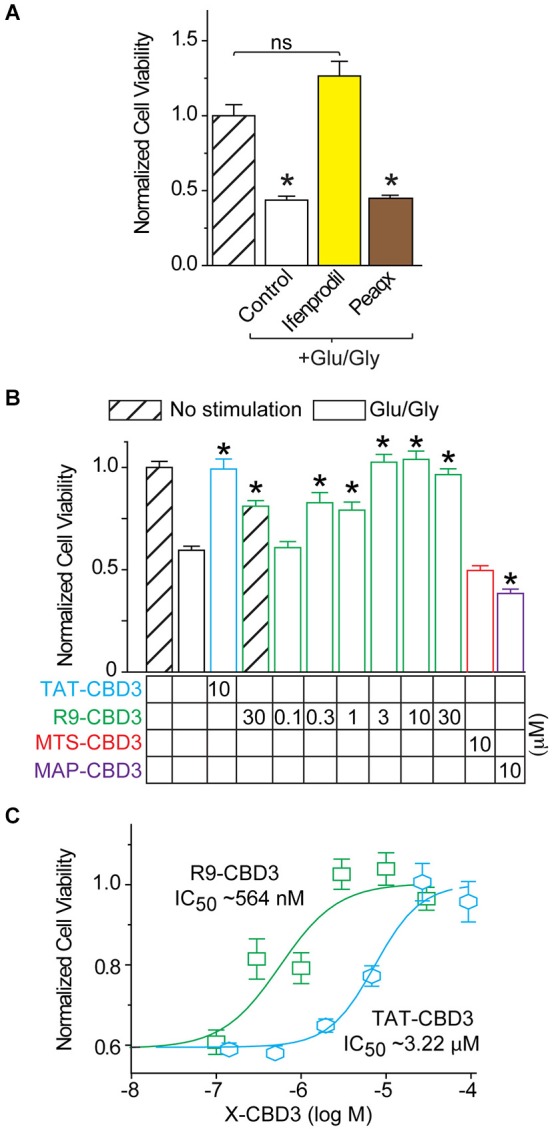
**R9-CBD3 is more potent than TAT-CBD3 in preventing glutamate-induced neuronal death. (A)** Cortical neurons were grown in culture for 7 DIV before being stimulated for 30 min with 200 µM glutamate and 20 µM glycine. Neurons were pre-incubated with either Ifenprodil or Peaqx for 10 min prior to stimulation and throughout the exposure to the excitotoxic insult. Values are the average of 32 wells from two separate experiments. Asterisk indicates significant difference compared to no stimulation control (*p < 0.05, one-way ANOVA with Dunnet’s *post hoc* test). **(B)** Cell viability of E18–19 DIV 7 cortical neurons was determined using a chromogenic cell viability assay. Neurons were incubated with peptides for 10 min prior to stimulation with 200 µM glutamate and 20 µM glycine for 30 min. Viability was then measured 24 h later, with all values normalized to no stimulation control (*n* = 32). **(C)** Concentration response viability curves for R9- and TAT-CBD3-treated neurons; values for R9-CBD3 are those shown in **(A)**. The IC_50_ of TAT- and R9-CBD3 was determined by fitting the curve to a four-parameter logarithmic function. Values are average of 32 wells from two separate experiments, asterisk indicates significant difference compared to stimulation in the absence of peptide. One-way ANOVA with *post hoc* Dunnett’s test indicated significant differences compared to control or peptide-treated groups; *p < 0.05.

As we observed inhibition of NMDAR-mediated Ca^2+^ influx and glutamate-induced DCD by several of the peptides, we next asked if this block of intracellular calcium escalation could potentially prevent glutamate-induce neurotoxicity. Because the NMDARs in cortical neurons at 7 DIV are primarily composed of NR2A and NR2B subunits, we first asked which of these subunits were being targeted in our studies. Furthermore, it had been previously reported that CRMP2 interacts with both subunits (Bretin et al., 2006). For these experiments, cortical neurons were pretreated for 10 min prior to stimulation with Ifenprodil (1 µM) and Peaqx (5 µM), specific blockers of the NR2B and NR2A subunits, respectively (Williams, 1993; Auberson et al., 2002) and also added throughout the stimulation phase of 200 µM glutamate and 20 µM glycine for 30 min. At 7 DIV it was found that Ifenprodil, but not Peaqx, was able to completely prevent Glu/Gly-induced neuronal cell death (Figure 6A). This finding suggests that NR2B is completely responsible for glutamate toxicity at this stage of culture, which is consistent with previous findings (Hardingham et al., 2002; Zhou and Baudry, 2006; Liu et al., 2007; Stanika et al., 2009). Next, neurons were incubated with CBD3 peptides conjugated with the four CPPs for 10 min prior to and throughout stimulation with Glu/Gly (Figure 6B). Cells were then fed with fresh conditioned media and grown for 24 h before cell viability was determined by a chromogenic cell viability assay. Consistent with previous findings (Brittain et al., 2011a), we observed an ~40% decrease in cell viability following glutamate challenge (Figure 6B). Incubation with TAT- or R9-CBD3 was completely neuroprotective as cell viability was not statistically different from control, unstimulated cells. Incubation with MTS-CBD3 had no effect on cell viability (0.49 ± 0.04, *n* = 16) while incubation with MAP-CBD3 appeared to be neurotoxic: cell viability 0.38 ± 0.01 (*n* = 32) compared to control 0.57 ± 0.06 (*n* = 22).

The neuroprotective efficacy of R9-CBD3 and TAT-CBD3 were determined by performing concentration-response curves using a range of concentrations for each peptide (Figure 6C). The concentration-response curves were fitted with variable Hill slope to generate an IC_50_ for each peptide. The extrapolated IC_50_ values for inhibition of glutamate-mediated toxicity were 564 nM and 3.2 µM for R9- and TAT-CBD3, respectively (Figure 6C). As neither MAP- nor MTS-CBD3 was neuroprotective, we did not test any additional concentrations for them.

Overall, these results are consistent with our earlier Ca^2+^-imaging experiments and demonstrate that TAT- and R9-CBD3 (i) reduce NMDAR-mediated Ca^2+^-influx; (ii) glutamate-induced DCD; and (iii) are neuroprotective. In contrast, MTS-CBD3 inhibits only glutamate-induced DCD while MAP-CBD3 is likely toxic as it induces an unchecked calcium influx in the absence or presence of an excitotoxic challenge (data not shown).

### Long-term application of MTS-CBD3 reduces glutamate toxicity

**Figure 7:**
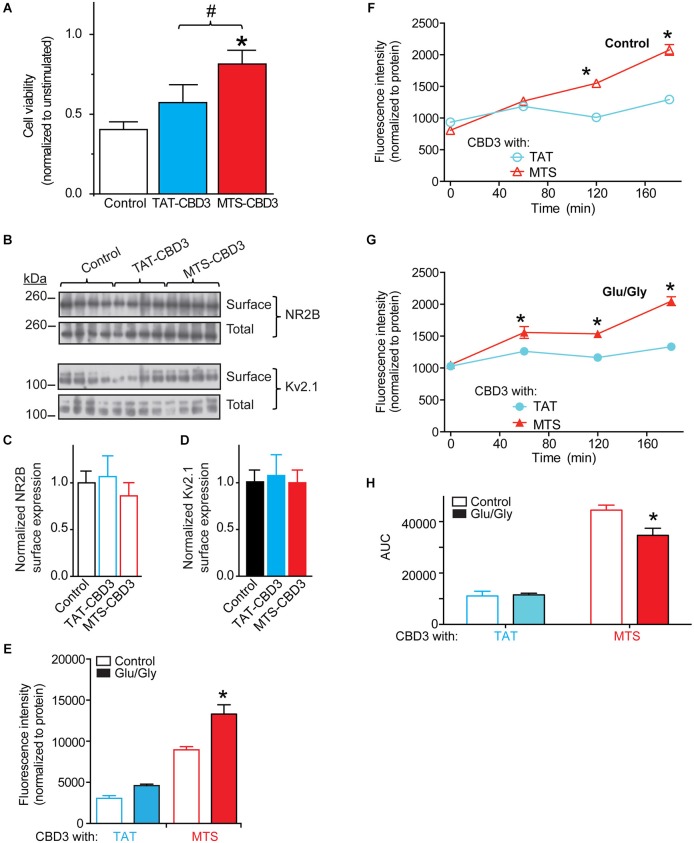
**Long-term application of MTS-CBD3 reduces glutamate-induced neuronal death likely due to increased intracellular retention of the peptide. (A)** Cortical neurons were treated with 10 µM of TAT- or MTS-CBD3 for 48 h prior to excitotoxic stimulation. Cell viability of peptide treated neurons was then measured 24 h following stimulation. Values for cell viability are normalized to unstimulated neurons treated with the same peptide. *p < 0.05 compared to vehicle treated neurons (Kruskal-Wallis non-parametric test with a Dunnett’s *post hoc* analysis). **(B)** Cortical neurons were treated with CBD3 peptides as in Figure 6 prior to labeling all surface exposed proteins using cell surface biotinylation. Representative immunoblots showing total and surface expressed NR2B along with the membrane bound Kv2.1. Average surface values from two separate neuron cultures with *n* = 13–15 for each condition. Values represent surface proteins divided by total proteins with all values normalized to the average for control neurons. Surface expression of NR2B **(C)** or Kv2.1 **(D)** is not affected by long term TAT- or MTS-CBD3 treatment. Cortical neurons plated onto poly-D-lysine-coated 96-well plates were incubated with FITC-labeled CBD3 peptides (20 µM) for 24 h min at 37°C, washed extensively with minimal essential media without phenol red, and fluorescence was measured 24 h later using a fluorescent plate reader at an excitation wavelength of 485 nm and emission wavelength of 520 nm. **(E)** Mean fluorescence uptake of peptides into cortical neurons, normalized to the amount of protein per well, was similar between untreated and Glu/Gly challenged neurons for TAT-CBD3 but higher in Glu/Gly challenged neurons compare to control neurons (*p < 0.01). Mean fluorescence efflux of peptides (chronic treatment) from untreated **(F)** or Glu/Gly-challenged **(G)** cortical neurons, normalized to the amount of protein per well, was significantly lower for TAT-CBD3 at 120 and 180 min compared to MTS-CBD3 (*p < 0.05). Additionally, the efflux at 1 h was also lower in the TAT-CBD3 vs. MTS-CD3-treated Glu/Gly-challenged neurons (*p < 0.05). Some error bars are smaller than the symbols. **(H)** AUC analyses reflecting cumulative efflux of the peptides in the indicated conditions. *p < 0.01 comparing AUC of total efflux for MTS-CBD3-treated control neurons vs. the Glu/Gly-challenged conditions. *n* = 2 separate, individual experiments; the total number of wells analyzed is 8–13 per condition.

Although neuroprotection was not conferred by an acute (10 min) exposure to MTS-CBD3, consistent with MTS-CBD3’s lack of inhibition of NMDAR-mediated Ca^2+^ influx, we postulated that due to its relatively long half-life, prolonged exposure to MTS-CBD3 may be effective in reducing glutamate-mediated neurotoxicity. We therefore treated neurons with 10 µM TAT-, MAP-, or MTS-CBD3 (R9-CBD3 was not tested due to its rather short *t*_1/2_ of 2 h) for 48 h prior to challenging them with an excitotoxic stimulus as described for Figure 6. Cell viability was normalized to unstimulated neurons treated for 48 h with the indicated peptides. In these experiments, MTS-, but not TAT-, CBD3 bestowed neuroprotection (Figure 7A). This suggests that MTS-CBD3 may have properties distinct from TAT-CBD3 or may remain active longer. Additional support for this is provided by data demonstrating that a 10 min incubation with 10 µM MTS-CBD3 was able to reduce, by ~75% compared to control, NMDA-mediated Ca^2+^ influx (see Figure 4E). MAP-CBD3-treated neurons did not survive the course of the experiment (viability less than <0.1 compared to untreated neurons; data not shown).

### Surface expression of NR2B is not affected by long term TAT- or MTS-CBD3 treatment

A potential mechanism for the observed neuroprotection by MTS-CBD3 may be via reduction of active NMDARs. Although as it appears that MTS-CBD3 does not acutely alter NMDAR-mediated Ca^2+^ influx it is still possible that chronic application of MTS-CBD3 may reduce surface expression of NMDARs. In order to test this hypothesis, neurons were treated as before with TAT- or MTS-CBD3 for 48 h and then surface NMDAR was quantified by cell surface biotinylation. Cell lysates (total) and biotin enriched samples (surface) were then immunoblotted for the NMDAR subunit NR2B (Figure 7B, *top blots*) and the unrelated surface membrane protein, the voltage-gated potassium channel Kv2.1 (Figure 7B, *bottom blots*). Surface expression of NR2B was normalized to total NR2B for each sample and then normalized to the average for control neurons (Figure 7C). We observed no difference in NR2B levels between neurons treated with either CBD3 peptide for 48 h and control neurons. Another surface protein, Kv2.1 was also tested and exhibited no changes following peptide (Figure 7D). This suggests that chronic MTS-CBD3 treatment reduces glutamate toxicity without altering NMDAR surface expression.

### Increased intracellular retention of MTS-CBD3

Another possibility that may account for the neuroprotection afforded by MTS-CBD3 may be differential influx and efflux propensities compared to TAT-CBD3. We tested this possibility by examining influx and efflux of fluorescent versions of TAT- and MTS-CBD3 peptides following a long-term application. Cortical neurons were incubated with 10 µM fluorescently labeled CBD3 peptides and the FITC fluorescence was determined. To reduce possible variability in uptake due to differences in cell plating, we normalized the fluorescence per well to the amount of protein. The fluorescence intensities were not different among between control- and Glu/Gly-treated neurons for TAT-CBD3, whereas the influx of MTS-CBD3 peptide was ~33% higher in cells challenged with Glu/Gly compared to control and ~65% higher than Glu/Gly-challenged cells incubated with TAT-CBD3 neurons (Figure 7E). We also sampled the media of these neurons to determine the extent of efflux of the fluorescently labeled peptides. As before, the fluorescence intensities were normalized to the amount of protein per well determined at the end of the experiment. At 1, 2 and 3 h post peptide application, the fluorescence intensities recorded for TAT- were lower than that for MTS-CBD3-treated cells irrespective of the excitotoxic challenge (Figures 7F,G). The cumulative efflux, calculated from the AUC analyses, was ~24% lower for MTS-CBD3-treated cortical cells exposed to a glutamate challenge compared to unchallenged cells, whereas TAT-CBD3-treated neurons had indistinguishable levels of efflux in control and challenged conditions (Figure 7H).

## Discussion

We previously identified CRMP2 as a novel target in NMDAR-mediated excitotoxicity (Brittain et al., 2011a, 2012). Targeting the function of CRMP2 with a 15 amino acid, cell penetrant peptide derived from the Ca^2+^ CBD3 of CRMP2, demonstrated neuroprotection both *in vitro* (glutamate induced excitotoxicity) as well as *in vivo* (middle cerebral artery occlusion and traumatic brain injury) (Brittain et al., 2011a, 2012). Furthermore, we demonstrated that coupling CBD3 to the hydrophilic charged TAT motif allowed the TAT-CBD3 peptide to enhance neuronal survival via direct inhibition of NMDARs (Brittain et al., 2011a). The goal of the present study was two-fold: to determine if coupling CBD3 to alternative cationic, hydrophobic, or amphipathic CPPs would result in (i) greater efficacy; and (ii) extended neuroprotection, compared to TAT-CBD3. Here, we report both greater efficacy and longer neuroprotection with CBD3 grafted to natural CPPs oligoarginine or the membrane translocating signal peptide sequence from the k-FGF receptor. Subunit diversity of NMDARs has been of keen interest in the field of excitotoxicity because the subunit composition of NMDARs appears to alter the extent of glutamate toxicity (Mizuta et al., 1998; Zhou and Baudry, 2006; Liu et al., 2007). Here, for the first time, we demonstrate that CBD3 peptides target only the NR2B isoform as ifenprodil, the NR2B selective drug, completely blocks glutamate toxicity in our cultures. In addition, our findings support the idea that tailoring of CPPs to the cargo may offer distinct advantages linked to the mechanism of action of the chosen CPP. A careful biochemical and functional examination of the CPP-cargo combination in *in vitro* experiments is warranted prior to selecting the best CPP-cargo pair for utility as promising signaling tools or therapeutic strategies *in vivo*.

### CRMP2, emerging roles in NMDAR-mediated excitotoxicity

Accumulating evidence suggests an important contribution of CRMP2 in glutamate-induced neurotoxicity. Lentiviral-mediated knockdown of CRMP2 reduced neuronal death following excitotoxicity (Brittain et al., 2011a) while increasing CRMP2 expression in axons made them resistant to toxic glutamate exposure (Hou et al., 2009). NMDAR activation leads to both proteolytic cleavage of CRMP2 by the calcium-activated protease calpain and phosphorylation of CRMP-2 at Thr-555 by Ca^2+^/calmodulin-dependent protein kinase II (CaMKII; Bretin et al., 2006; Hou et al., 2009). Of further interest, it appears that phosphorylation of CRMP2 may protect it from being cleaved. This presents a potentially complex pathway where CaMKII activation and subsequent phosphorylation prevents concomitant calpain cleavage of CRMP2. CaMKII-mediated phosphorylation of CRMP2 is predicted to cause a reduction in axon growth potential through a reduced affinity of CRMP2 for tubulin and Numb (Arimura et al., 2005). Perhaps more intriguing is that it was observed in this study that overexpression of CRMP2 prevented glutamate induced alteration of neuritic processes, while a T555A mutant had no effect (Hou et al., 2009). While this suggests that Thr-555 phosphorylation site is not an important determinant in neuroprotection, it is at present unknown, what effect, if any cleavage of CRMP2 may have on neuronal survival. Alternatively, it has been shown that a calpain-cleaved version of CRMP-2 is neuroprotective when overexpressed in neurons, possibly via downregulating NMDAR responses (Bretin et al., 2006). This finding is difficult to interpret, however, because expression of a similarly truncated CRMP2 leads to a reduction in neuritic process growth, which can likely affect many pathways responsible for glutamate toxicity (Rogemond et al., 2008). It is more likely that expression of this truncated form of CRMP2 acts as a dominant negative. This could occur either through sequestering native CRMP2 (through tetramerization) or competing with native CRMP2 for endogenous protein interactions required for neuritic outgrowth (e.g., tubulin and actin). Therefore, the neuroprotection observed following knockdown of CRMP2 is entirely consistent with the neuroprotection conferred by the calpain-cleaved form of CRMP2, in that loss of CRMP2 function observed with the latter construct is neuroprotective (Bretin et al., 2006). It is then perhaps no surprise that over-expression of full length CRMP2 enhances neurotoxicity (Bretin et al., 2006).

### TAT-CBD3, a neuroprotective peptide

A short fragment of CRMP2 (CBD3) coupled to the charged CPP motif TAT, was neuroprotective (Brittain et al., 2011a). The *modus operandi* of TAT-CBD3 involved: (i) attenuation of Ca^2+^-influx through NMDARs; (ii) reduction of calpain-mediated cleavage of CRMP2; and (iii) induction of internalization of dendritic NMDARs resulting in sparing of neurons following an excitotoxic insult (Brittain et al., 2011a). A similar TAT-conjugated CRMP2 peptide overlapping somewhat with the CBD3 region of CRMP2 (i.e., TAT-CRMP2, amino acids 492–506 vs. CBD3, amino acids 484–499) also demonstrated efficacy in reducing infarct volume associated with a cerebral ischemic injury (Yin et al., 2013). Here, we extended the mechanism of action of TAT-CBD3 by providing evidence of direct inhibition of the interaction between the NR2B-NMDAR and CRMP2. Additionally, we demonstrated that TAT-CBD3, while effective in preventing glutamate-induced cell death when applied for an acute period (10 min prior to the onset of the glutamate challenge) is ineffective when applied for longer (~48 h prior to the onset of the glutamate challenge); the lack of effect likely stems from the relatively short half-life (~9 h) of TAT (Sarko et al., 2010) coupled with the lack of intracellular retention as its amount of efflux was doubled under excitotoxic conditions (Figures 7E,H).

### R9-CBD3, an acutely acting neuroprotective peptide

**Figure 8:**
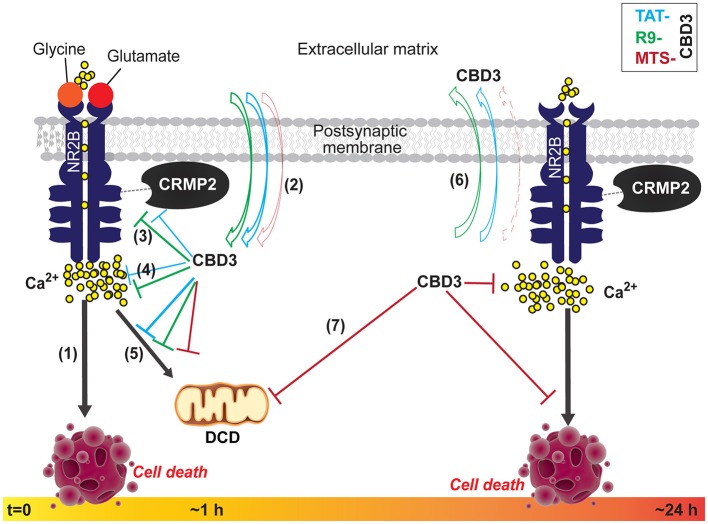
**Summary of the biochemical and functional effects of CBD3 peptides conjugated to cationic, hydrophobic, and amphipathic CPPs in neuroprotection. (1)** Following obligatory binding of glutamate and glycine to their cognate sites on the extracellular face of the NMDAR, an influx of Na^+^ and Ca^2+^ ions ensue. Hyperactivation of NMDARs, such as that seen in injury, leads to a substantial influx of Ca^2+^ ions that leading to the over-activation of several deleterious enzymes and signaling pathways that harm neurons or lead to cell death (neurotoxicity). We previously demonstrated that TAT-CBD3, a CRMP2 derived peptide, mitigates Ca^2+^-influx through NMDARs and consequently is neuroprotective (Brittain et al., 2011a). **(2)** With acute application, CBD3 conjugated to other cationic (R9 or MTS) or amphipathic (MAP) cell penetrating peptide (CPP) motifs differentially enters neurons with more R9-CBD3 than TAT-CBD3 exiting the membrane under conditions when the neurons are faced with a glutamate challenge. **(3, 4)** Both TAT- and R9-CBD3 blunt the interaction between CRMP2 and the NR2B-NMDR as well as cause inhibition of NMDA-mediated Ca^2+^-influx, with R9-CBD3 being more potent than TAT-CBD3 in both events. **(5)** Following exposure (~1 h) to a glutamate challenge, the neurons swell with Ca^2+^, likely due to a failure in the ability of mitochondria to buffer Ca^2+^, leading to the phenomenon of delayed Ca^2+^ deregulation (DCD) which precedes the necrotic death of neurons; TAT-, R9-, and MTS-CBD3 prevent this calcium rise by blunting DCD likely leading to preservation of neuronal viability. **(6)** The peptides, applied for a longer period (>24 h), exhibit different behaviors vs. short-term applications with a greater net accumulation of MTS-CBD3 compared to TAT-CBD3. **(7)** Where long-term TAT-CBD3 treatment is ineffective in protecting neurons from glutamate-induced neurotoxicity and cell death, MTS-CBD3 preserves neuronal viability while also inhibiting DCD. Collectively, these findings suggest that tailoring CBD3 peptides with alternative CPPs, such as MTS, results in extended neuroprotection beyond that of TAT-CBD3.

Arginine rich CPPs, and R9 in particular, have been reported to display excellent cell penetrating abilities (Wender et al., 2000). This CPP uses a combination of macropinocytosis, clathrin-mediated endocytosis and caveolae-dependent endocytosis for uptake into cells (Duchardt et al., 2007). Of the CPPs tested in this study, R9 had the shortest half-life (Sarko et al., 2010). Attaching R9 to CBD3 yielded a peptide that was more effective than TAT-CBD3 in reducing NMDAR-mediate Ca^2+^-influx, likely due to an enhanced inhibition of the NR2B-NMDAR and CRMP2 interaction when compared to TAT-CBD3. R9-CBD3, like TAT-CBD3, was neuroprotective; however, R9-CBD3 was ~6-fold more potent than TAT-CBD3. Inhibition of glutamate-trigged DCD was also inhibited by R9-CBD3 implying a neuroprotective mechanism similar to that of TAT-CBD3 (Figure 8).

### MTS-CBD3, a long-acting neuroprotective peptide with a novel mechanism of action

Membrane translocating peptides (MTS) are largely hydrophobic and typically originate from secretory proteins that translocate through cellular membranes. The translocating potential of the MTS CPP seems to be predicated on its overall hydrophobic composition. Appending the 16-residue sequence from the signal peptide of the Kaposi fibroblast growth factor (k-FGF) to CBD3 bestowed upon the MTS-CBD3 peptide an ability to be neuroprotective for neurons pre-treated with MTS-CBD3 for 48 h prior to excitotoxic stimulation. This salient finding distinguishes this version of the CBD3 peptide from R9- and TAT-CBD3, which are neuroprotective only acutely. While the long-term neuroprotection may be explained by the long half-life (~48 h) (Sarko et al., 2010) of MTS, additional differences may also contribute. The mechanism of how MTS-CBD3 achieves long-term neuroprotection also seems to differ from the other CBD3-CPP combinations tested here in that the MTS-CBD3 peptide does not affect the interaction between the NR2B-NMDAR and CRMP2, nor does it affect surface trafficking of NMDARs. A more likely explanation for the longer neuroprotection is an increased retention of the MTS-CBD3 peptide, compared to TAT-CBD3, following conditions of glutamate challenge. While the MTS-CBD3 peptide did not abrogate the NR2B-NMDAR and CRMP2 interaction, it did prevent NMDAR-mediated Ca^2+^-influx as well as blocked glutamate-induced DCD (Figure 8), suggesting the possible involvement of other protein(s). While not investigated in the present study, we recently reported that TAT-CBD3 inhibited increases in cytosolic Ca^2+^ mediated by the plasmalemmal Na^+^/Ca^2+^ exchanger (NCX) operating in both the forward and reverse modes (Brustovetsky et al., 2014). Whether MTS-CBD3 affects NCX similarly is currently unknown. The translocation of MTS is believed to occur directly through the lipid bilayer. Both membrane fluidity and lateral mobility of membrane proteins influence the translocation process and the α-helical conformation formed by the MTS sequence facilitates membrane interactions. CBD3 has been modeled as an α-helix (Piekarz et al., 2012). The combined MTS-CBD3 peptide, if it adopts an α-helical conformation, may remain tethered to the membrane thus accounting for the increased retention and perhaps also be relatively spared from proteases that may otherwise degrade and inactivate the peptide; the latter may explain the long term neuroprotective effects as well. The lack of inhibition of the NR2B-NMDAR and CRMP2 interaction by MTS-CBD3 may possibly be due to an insufficient amount of CBD3 available for interfering with this complex in the cytosol. That MTS-CBD3 did not inhibit the NR2B-NMDAR and CRMP2 complex may explain the seemingly incongruous results of inhibition of NMDA-mediated Ca^2+^ influx following a 10 min application between NMDA pulses (Figure 4E) without a commensurate neuroprotection in the short-term (10 min) application (Figure 6B). In other words, it is plausible that due to its likely association with the membrane, MTS-CBD3 may be in proximity of NMDARs and thus be able to block Ca^2+^ influx but without a long incubation (>24 h), an insufficient amount gets into the cells to be able to block cell death.

### Toxic consequences of MAP-CBD3

It was previously demonstrated that the localization pattern of the amphipathic peptide, MAP, is largely nuclear in contrast to the predominantly cytosolic targeting of the cationic CPPs oligoarginine and TAT (Zaro et al., 2009). The authors also noted high accumulation of MAP within the nuclei and nuclear membrane as well as in intracellular vesicles at the periphery of the nucleus (Zaro et al., 2009). Notably, a truncated form of CRMP2 missing its 69 carboxyl terminal residues is also localized to the nucleus; the nuclear targeting of CRMP2 is provided by a nuclear localization signal within residues Arg 471 and Lys 472 in its primary sequence (Rogemond et al., 2008). Overexpression of this truncated form of CRMP2 in cortical neurons improves their resistance to NMDA cytotoxicity (Bretin et al., 2006). It is plausible that MAP-CBD3, by virtue of its intrinsic nuclear targeting, interferes with this CRMP2-mediated resistance in the face of excitotoxicity. In support of this assertion are findings which demonstrate that, in HeLa cells, the truncated CRMP2 form hastens apoptotic nuclei and cell death (Tahimic et al., 2006). The rapid (within 2 min) increase in Ca^2+^ influx observed in our experiments following treatment of cortical neurons with MAP-CBD3 (data not shown), coupled with possible interference of CRMP2 nuclear targeting, may account for the complete failure of MAP-CBD3 in neuroprotection. It has also been reported that some primary amphipathic CPPs are toxic to cells even at low concentrations (Madani et al., 2011).

## Conclusions

The data presented here support the idea that the neuroprotective function of CBD3 may be temporally segregated into acute (with R9 or TAT) or chronic (with MTS) modalities. The novel CBD3-CPP combinations reported here, namely R9-CBD3 and MTS-CBD3, may serve as useful tools in dissecting early vs. late biochemical events, respectively, in NMDAR-mediated signaling leading to excitotoxicity and cell death (Figure 8). Importantly, the data also underscore the importance for any strategy involving the use of CPPs to deliver bioactive cargo of first testing a diverse set of CPPs prior to their translation *in vivo*.

## Authors and contributors

Participated in Research Design—Aubin Moutal, Liberty François-Moutal, Joel M. Brittain, May Khanna, Rajesh Khanna. Conducted Experiments—Aubin Moutal, Liberty François-Moutal, Joel M. Brittain, May Khanna, Rajesh Khanna. Performed Data Analysis—Aubin Moutal, Liberty François-Moutal, Joel M. Brittain, Rajesh Khanna. Wrote the Manuscript—Aubin Moutal, Rajesh Khanna.

## Conflict of interest statement

The authors declare that the research was conducted in the absence of any commercial or financial relationships that could be construed as a potential conflict of interest.

## Abbreviations

AUC: area under the curve
CBD3: Ca^2+^ channel binding domain 3
CPPs: cell penetrating peptides
CRMP2: collapsin response mediator protein 2
DCD: delayed Ca^2+^ deregulation
DIV: days *in vitro*
E19: embryonic day 19
HIV1: human immunodeficiency virus type 1
MAP: model amphipathic peptide
MTS: membrane translocating sequence of Kaposi fibroblast growth factor receptor
NMDARs: *N*-methyl-D-aspartate receptors
NR2B: NMDAR subunit 2B
PTD: protein transduction domain
R9: nona-arginine
TAT: transactivator of transcription domain.
